# Analysis of metabolic pathways related to fertility restoration and identification of fertility candidate genes associated with *Aegilops kotschyi* cytoplasm in wheat (*Triticum aestivum* L.)

**DOI:** 10.1186/s12870-019-1824-9

**Published:** 2019-06-11

**Authors:** Sha Li, Zihan Liu, Yulin Jia, Jiali Ye, Xuetong Yang, Lingli Zhang, Xiyue Song

**Affiliations:** College of Agronomy, Northwest A&F University, Yangling, Shaanxi China

**Keywords:** Comparative transcriptome, Cytoplasmic male sterility, Flavanone 3-hydroxylase, Pectin methylesterase, Wheat (*Triticum aestivum* L.), WRKY transcription factor

## Abstract

**Background:**

Thermo-sensitive male-sterility based on *Aegilops kotschyi* cytoplasm (K-TCMS) plays an important role in hybrid wheat breeding. This has important possible applications in two-line hybrid wheat breeding but the genetic basis and molecular regulation mechanism related to fertility restoration are poorly understood. In this study, comparative transcriptome profiling based on RNA sequencing was conducted for two near-isogenic lines comprising KTM3315R and its sterile counterpart KTM3315A, a total of six samples (3 repetitions per group), in order to identify fertility restoration genes and their metabolic pathways.

**Results:**

In total, 2642 significant differentially expressed genes (DEGs) were detected, among which 1238 were down-regulated and 1404 were up-regulated in fertile anthers. Functional annotation enrichment analysis identified important pathways related to fertility restoration, such as carbohydrate metabolism, phenylpropanoid metabolism and biosynthesis, as well as candidate genes encoding pectin methylesterase and flavanone 3-hydroxylase. Moreover, transcription factor analysis showed that a large number of DEGs were mainly involved with the WRKY, bHLH, and MYB transcription factor families. Determination of total soluble sugar and flavonoid contents demonstrated that important metabolic pathways and candidate genes are associated with fertility restoration. Twelve DEGs were selected and detected by quantitative reverse-transcribed PCR, and the results indicated that the transcriptome sequencing results were reliable.

**Conclusions:**

Our results indicate that identified DEGs were related to the fertility restoration and they proved to be crucial in *Aegilops kotschyi* cytoplasm. These findings also provide a basis for exploring the molecular regulation mechanism associated with wheat fertility restoration as well as screening and cloning related genes.

**Electronic supplementary material:**

The online version of this article (10.1186/s12870-019-1824-9) contains supplementary material, which is available to authorized users.

## Background

Heterosis is one of the most successful strategies for increasing crop yields and it has been exploited widely in plant breeding systems [1]. In this context, cytoplasmic male sterility (CMS) can create sterile male gametophytes without affecting the agronomic performance and this cost-effective system facilitates hybrid seed production [2, 3]. In plants, CMS is due to the maternally inherited inability to establish genome coordination between the organelles (mitochondria) and nuclear genomes, thereby resulting in non-functional pollen production [4–7]. Due to the CMS trait, the requirement for the manual removal of anthers can be eliminated, thereby reducing the needs in terms of labor and material resources, as well as allowing the hybridization technique to produce excellent F_1_ generations that are significantly superior to the parent and available popular breeds in terms of their yield, stress resistance, and adaptability [8]. CMS has been applied in crops such as rice [9], maize [10], sorghum [11], soybean [12], and cotton [13] with remarkable results. However, due to its huge genome and long breeding cycle, the use of CMS in wheat production is still a challenge.

The use of CMS in wheat has important implications for simplifying breeding programs and increasing crop yields and quality. At present, the thermo-sensitive CMS wheat lines with *Aegilops kotschyi* cytoplasm (K-TCMS) based on the two-line method have advantages as simple restorer and maintenance lines, with no negative cytoplasmic effects [14]. These lines perform well in hybrid wheat breeding and they can produce a large number of hybrid seeds, which is a valuable benefit for the production of hybrid wheat [15, 16].

In recent years, high-throughput sequencing has provided a new approach for transcriptome sequencing and it is especially helpful for conducting comparative analyses of RNA sequencing data obtained from non-model species with limited genomic information. Comparative transcriptome analysis has been successfully employed to study the molecular mechanism of male sterility at different developmental stages in different species. For example, the candidate genes and important metabolic pathways have been studied in cotton [17, 18], *Brassica napus* [19, 20], rice [21], cabbage [22], sesame [23], and other species. However, few comparative transcriptome studies of fertility restoration in wheat have been conducted.

In order to improve the utilization of wheat heterosis and facilitate agricultural development, it is important to elucidate the molecular mechanism responsible for fertility restoration in CMS lines. In the present study, anthers from the binucleate stage of the K-TCMS wheat line KTM3315A and its near-isogenic restoring line KTM3315R were used as experimental materials for RNA sequencing to identify important candidate genes and the biological pathways related to fertility restoration. In particular, we aimed to: obtain novel insights into the molecular regulation mechanism associated with fertility restoration in wheat; and provide a foundation for screening and further cloning genes related to fertility restoration in subsequent studies.

## Results

### Phenotypic traits and microscopic observations

**Fig. 1:**
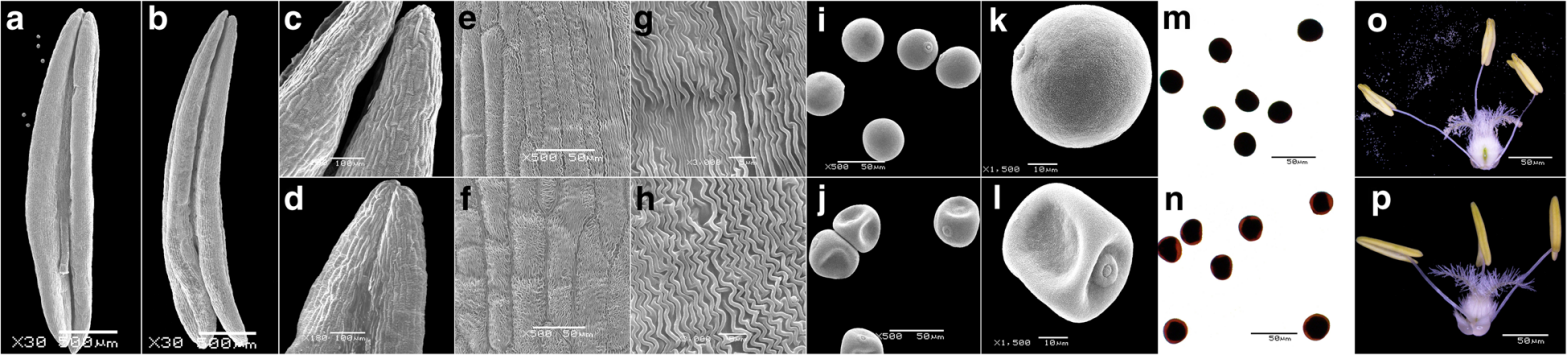
Scanning electron microscopy images of KMF (**a**, **c**, **e**, **g**, **i**, **k**) and KMA (**b**, **d**, **f**, **h**, **j**, **l**). I_2_–KI staining of KMF (**m**) and KMA (**n**). Dissection microscopy observations of KMF (**o**) and KMA (**p**). Anthers (**a**–**d**, **o**, **p**). Anther outer epidermis (**e**–**h**). Trinucleate stage microspores (**i**–**n**). Scale bars = 500 μm (**a**, **b**); 100 μm (**c**, **d**); 50 μm (**e**, **f**, **i**, **j**, **m**–**p**); 10 μm (**k**, **l**); 5 μm (**g**, **h**)

In order to determine the visual differences between the fertile and sterile wheat lines, we observed the morphology of anthers, anther outer epidermis, and microspores in the fertile wheat line KTM3315R and sterile wheat line KTM3315A during the trinucleate stage (Fig. 1). Scanning electron microscopy showed that, compared with the KTM3315A anthers, the KTM3315R anthers were plump, symmetrical, and regular, and the upper ends of the anthers were cracked and they released mature pollen grains (Fig. 1a–d). Dissection microscopy also clearly demonstrated these features (Fig. 1o, p). Observations of the outer epidermis of the anthers (Fig. 1e–h) showed that the folds formed by the KTM3315R anthers were regular and flat (Fig. 1g), whereas they were irregular in KTM3315A (Fig. 1h). In addition, the mature pollen grains of KTM3315A were shrunken and deformed, whereas those of KTM3315R were plump and spherical (Fig. 1i–l). Cytological observations of the mature pollen grains were also obtained by I_2_–KI staining, which showed that the mature pollen grains stained completely black in KTM3315R, whereas the mature pollen grains at the periphery were not stained in KTM3315A and the staining was uneven, which is the typical staining pattern for abortive pollen (Fig. 1 m, n). According to these observations, there were clear differences in the fertility phenotype between KTM3315A and KTM3315R due to the induction of fertility restoration genes.

### Transcriptome sequencing and genome mapping

Based on the phenotypic traits observed for the anthers and mature pollen grains, we hypothesized that the gene expression pattern had changed. To validate our hypothesis and further elucidate the molecular mechanisms of regulating fertility, we employed an Illumina HiSeq PE150 sequencer for high-throughput sequencing of the fertile and sterile anthers from KTM3315R (designated as KMF) and KTM3315A (designated as KMA), a total of six samples (3 repetitions per group, including KMA1, KMA2, KMA3; KMF1, KMF2, KMF3. respectively), at the binucleate stage.

**Fig. 2:**
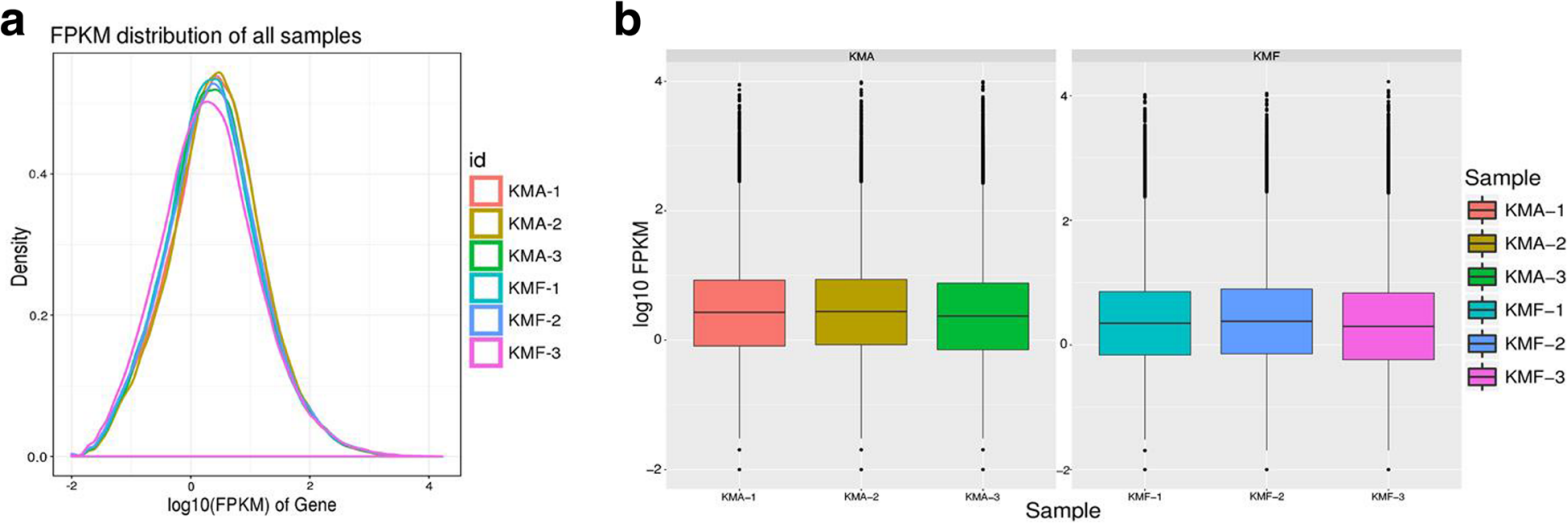
FPKM density and box plot FPKM distributions for each sample. The different colored curves denote different samples. The horizontal axis indicates the FPKM for the corresponding sample and the vertical axis indicates the corresponding probability density (**a**). The horizontal axis represents different samples and the vertical axis represents the FPKM for samples (**b**). KMA comprises three biological repeats for KMA-1, KMA-2, and KMA-3. KMF comprises three biological repeats for KMF-1, KMF-2, and KMF-3

In total, 44 GB of clean data were produced. The data were filtered for each sample and an average of 7.3 GB of high quality clean data was obtained where the Q20 (sequencing error rate, 1%) base percentage exceeded 95.69%. All of the bases were identified and the CG content ranged from 59.15 to 61.62%. The average read length of each sequence was 150 bp. For each sample, the high quality clean reads were filtered using the rRNA database and aligned with the reference genome, where the alignment efficiency was between 63.11 and 68.59% (Additional file 1: Table S1). After directly comparing the abundances and discrete distributions of the expression levels for different sample genes, we found that the sequencing quality and gene expression levels were basically the same (Fig. 2a, b). Thus, the results indicated that the quality of the data obtained by sequencing was reliable and suitable for subsequent analyses.

### Identification of DEGs

**Fig. 3:**
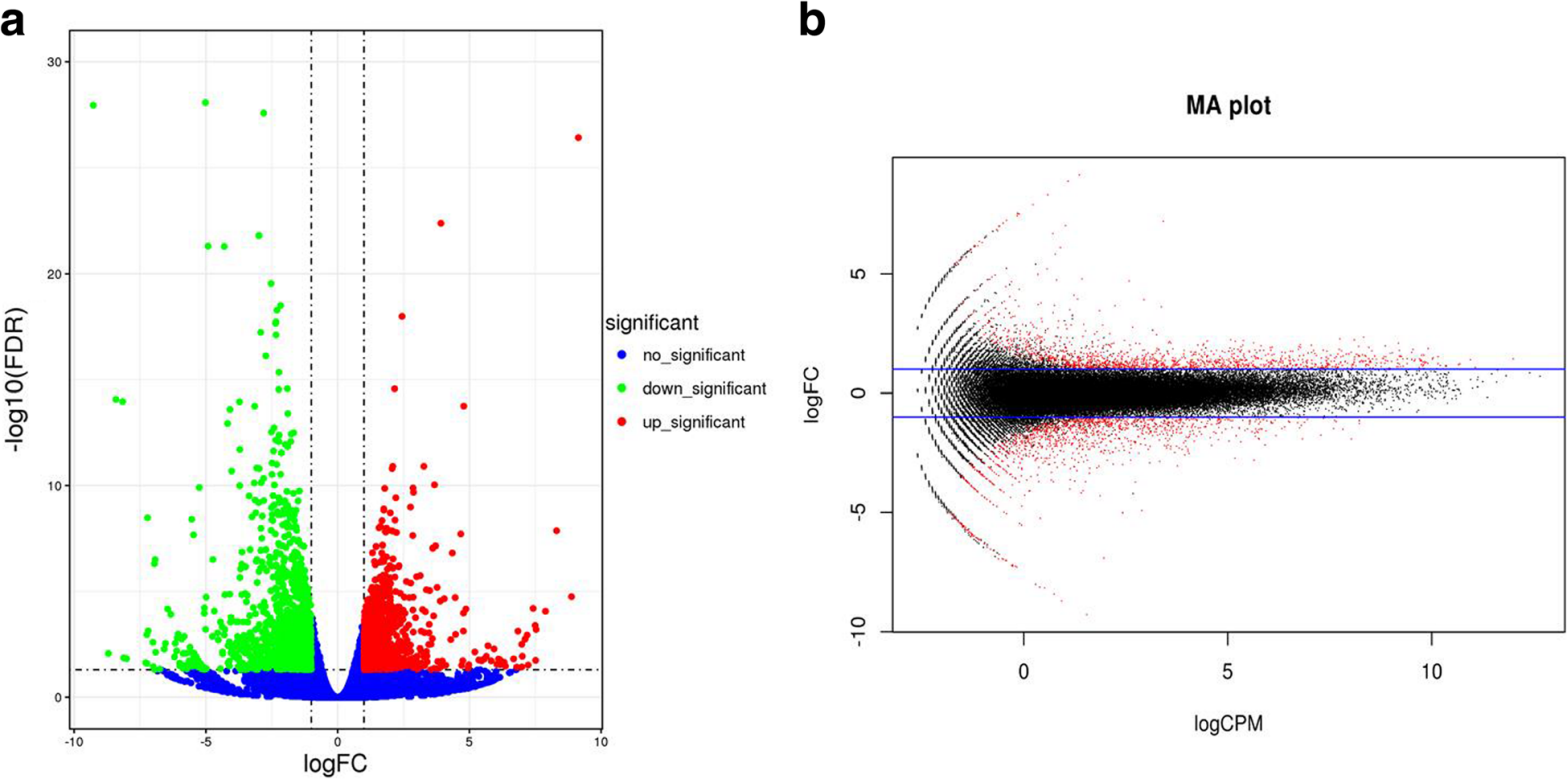
Volcano plot and smear diagram of the differential gene expression levels in KMF and KMA. Green indicates downregulation in KMF and red indicates upregulation in KMF (**a**). The abscissa is the base 10 logarithm of counts per million (CPM) and the ordinate is the base 2 logarithm of the fold change (FC) (**b**)

To identify differentially expressed genes across groups, the edgeR package was used [24]. We identified genes with log_2_(fold change (FC) ≥ 1 and the false discovery rate (FDR) < 0.05 in a comparison as significant DEGs. In total, 2642 DEGs were identified in the KMF and KMA groups (Additional file 2: Table S2), where 1238 were down-regulated and 1404 were up-regulated in KMF compared with KMA. We used a volcano plot and a smear diagram to visualize the significant differences in DEGs (Fig. 3a, b). The subsequent bioinformatics analyses were conducted based on these DEGs.

### Functional classification of DEGs

**Fig. 4:**
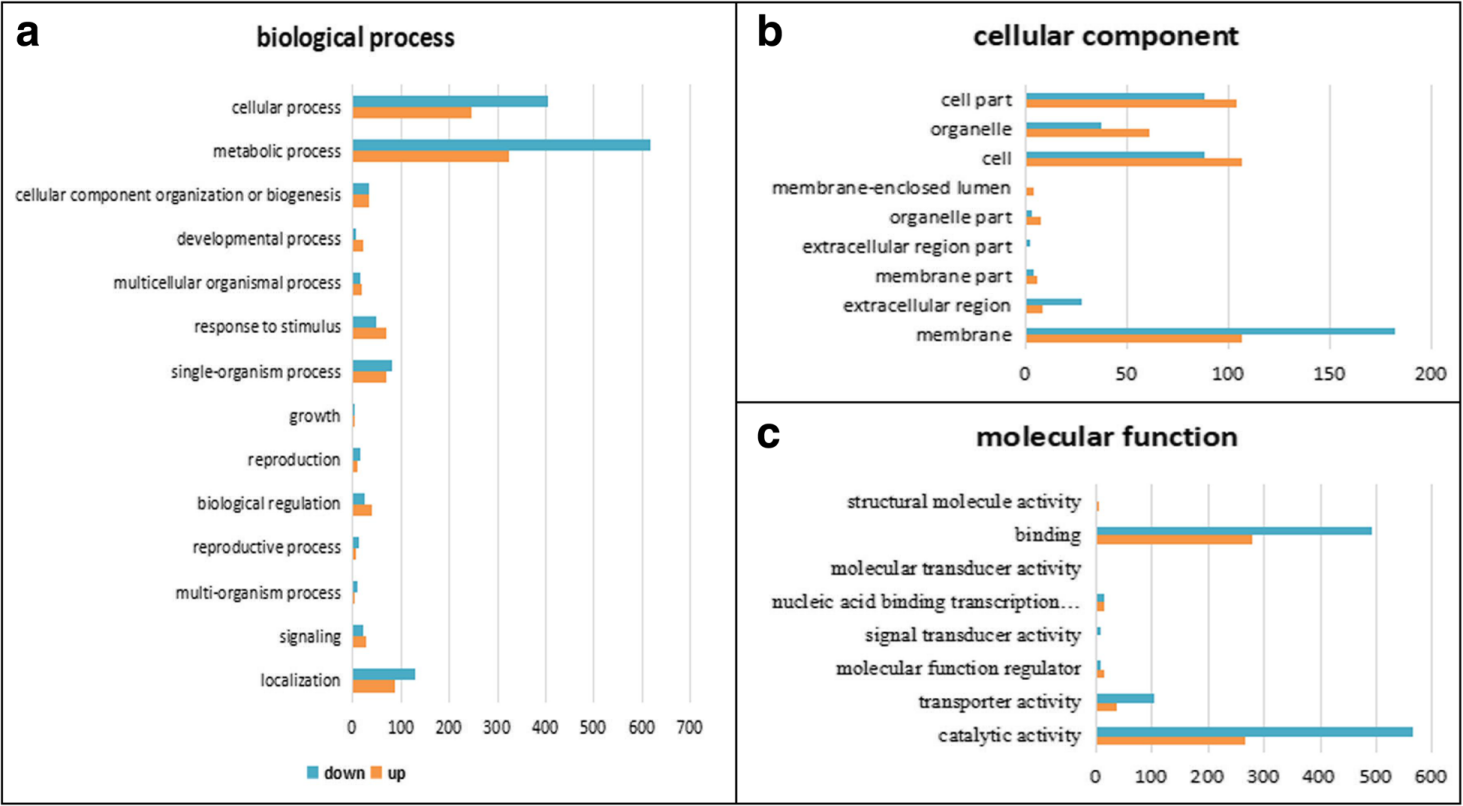
Gene Ontology (GO) classifications of differentially expressed genes (DEGs) in KMF and KMA. Biological process (**a**). Cellular component (**b**). Molecular function (**c**). The horizontal axis shows the number of enriched DEGs in each main category and the vertical axis represents each GO term. “Up” denotes upregulated DEGs and “down” represents downregulated DEGs. For each GO term, the first column shows the number of downregulated DEGs and the second column on shows the number of upregulated DEGs

To determine the major biological functions of differentially expressed genes, 2642 DEGs were sequentially aligned into the Gene Ontology (GO), Eukaryotic Orthologous Groups (KOG), and Kyoto Encyclopedia of Genes and Genomes (KEGG) databases. Using the hypergeometric test, the q-value≤0.05 obtained after the *P*-value is corrected by multiple tests is used as the threshold, and the GO term and path which are significantly enriched in the DEGs are obtained. Due to the large numbers and the complex branch structure of GO categories, only the three most abundant functional groups, namely “Biological Process”, “Cellular Component” and “Molecular Function” were presented, as an example (Fig. 4a-c, Additional file 3: Table S3-1). In the sub-category of “Biological Process”, “metabolic process (GO: 0008152)” and “cellular process (GO: 0009987)” were the most abundant cascades that have a respective of 942 and 649 DEGs. In these two sub-categories, “catabolic processes (GO: 0009056)”, “secondary metabolic processes (GO: 0019748)” and “cell death (GO: 0008219)” were significantly enriched (Additional file 3: Table S3–2). In the next main sub-category of “Cellular Component”, “membrane (GO: 0016020)” and “cell (GO: 0005623)” were the most abundant cascades that have a respective of 288 and 194 DEGs, respectively. In the “cell” category, DEGs were significantly enriched for “cell wall (GO: 0005618)” (Additional file 3: Table S3–2). Within the last sub-category “Molecular Function”, “catalytic activity (GO:0003824)” and “binding (GO:0005488)” were the most abundant cascades that have 833 and 771 DEGs, respectively, where the number of down-regulated DEGs was almost two times that of up-regulated DEGs. In these two sub-categories, “kinase activity (GO: 0016301)”, “hydrolase activity (GO: 0016787)” and “Carbohydrate binding (GO: 0030246)” were significantly enriched (Additional file 3: Table S3–2). Based on these significantly enriched terms, we predict that they are closely related to the fertility restoration of wheat.

**Fig. 5:**
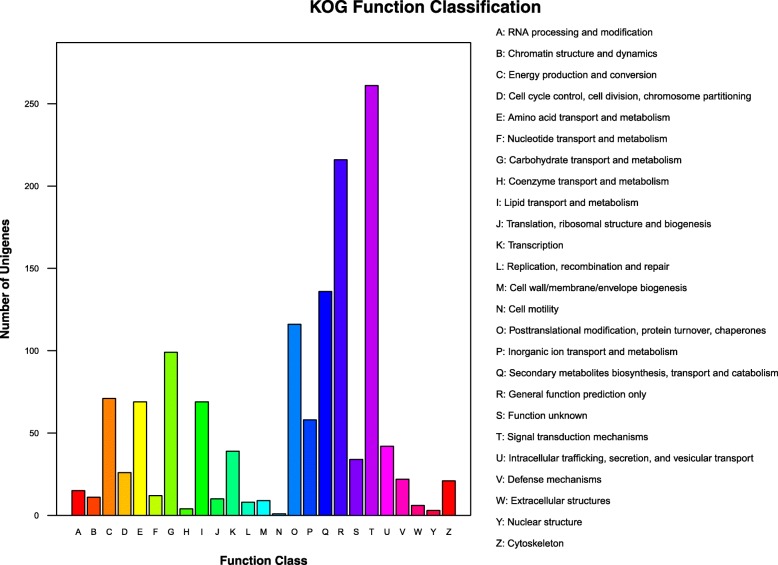
Eukaryotic Orthologous Groups (KOG) protein classifications for differentially expressed genes (DEGs) in KMF and KMA. Capital letters on the x-axis represent the KOG categories listed to the right of the histogram. The y-axis shows the number of DEGs

According to the KOG annotated results, 1198 DEGs were annotated into 25 KOG categories (Fig. 5, Additional file 4: Table S4). “Signal transduction mechanisms” was the largest group (group T with 261 DEGs), followed by “General function prediction only” (group R with 216 DEGs) and “Secondary metabolites biosynthesis, transport and catabolism” (group Q with 136 DEGs). “Post-translational modification, protein turnover, chaperones” (group O with 116 DEGs), “Carbohydrate transport and metabolism” (group G with 99 DEGs), “Energy production and conversion” (group C with 71 DEGs), “Amino acid transport and metabolism” (group E with 69 DEGs), and “Lipid transport and metabolism” (group I, with 69 DEGs) were the other main categories. Therefore, the eight categories highlighted above may be important for understanding fertility.

**Fig. 6:**
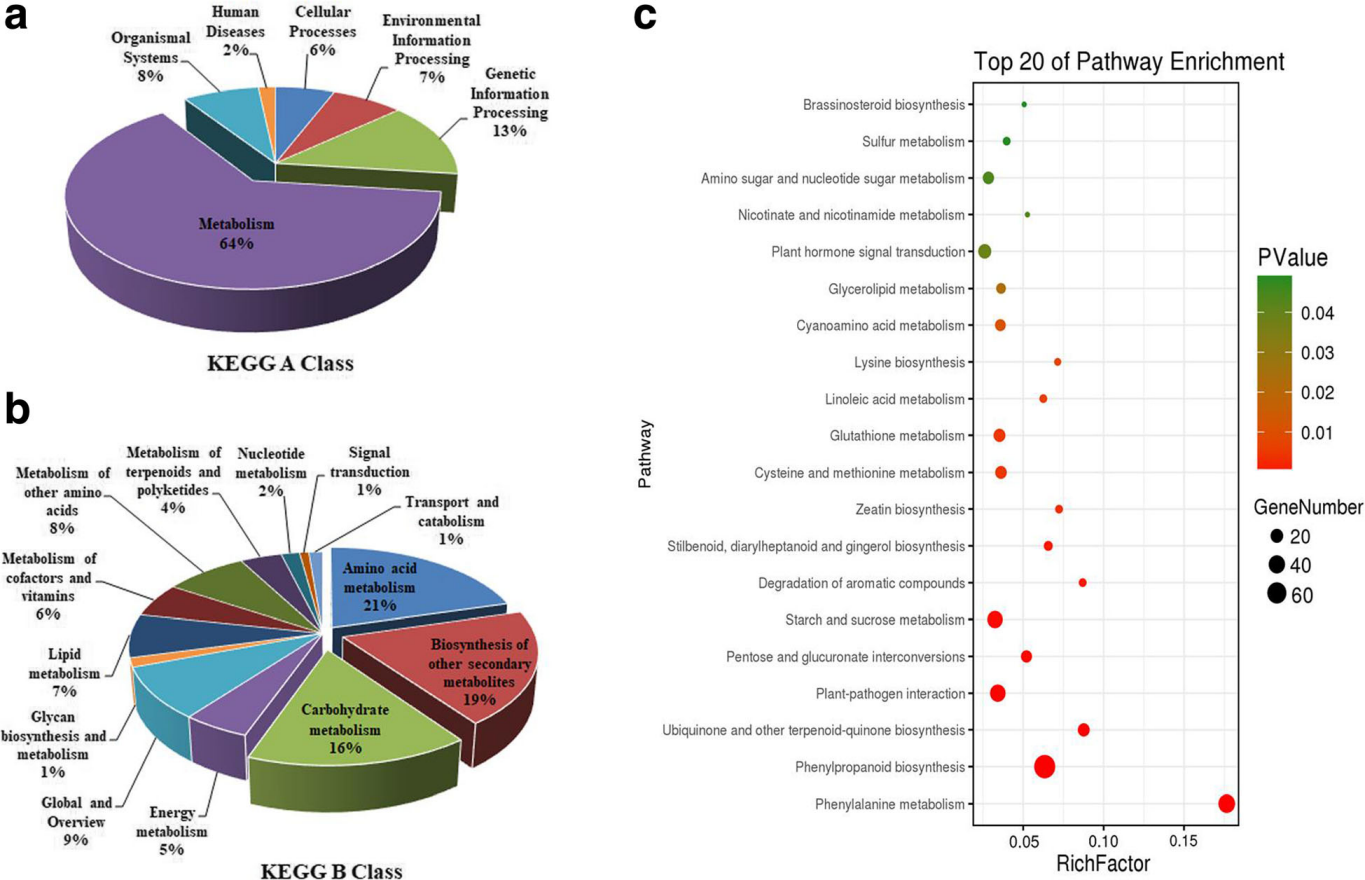
KEGG pathway classifications. **a** KEGG A Class. **b** KEGG B Class. **c** Differential gene enrichment bubble chart. X-axis label: enrichment factor. Y-axis label: pathway name. Bubble area size: number of genes in the target gene set that belong to a pathway. Bubble color: enriched in terms of significance according to the p-value

In the KEGG analysis, a total of 438 DEGs were annotated and classified into 102 pathways. Metabolism (64%, 296 DEGs) in classification A categories accounted for most of the DEGs, followed by B categories, where they were mainly enriched in terms of amino acid metabolism (21%, 93 DEGs), biosynthesis of other secondary metabolites (19%, 87 DEGs), and carbohydrate metabolism (16%, 71 DEGs) (Fig. 6a, b). In the amino acid metabolism category, phenylalanine metabolism (ko00360) and cysteine and methionine metabolism (ko00270) were enriched most with 42 and 16 DEGs, respectively. In the biosynthesis of other secondary metabolites category, phenylpropanoid biosynthesis (ko00940) was enriched most with 77 DEGs. In the carbohydrate metabolism category, significant enrichment was determined for starch and sucrose metabolism (ko00500) and pentose and glucuronate interconversion (ko00040) with 34 and 14 DEGs, respectively. A high-level bubble diagram was prepared for the top 20 pathways where *p* ≤ 0.05 in order to visualize the significant pathways (Fig. 6c, Additional file 5: Table S5). Based on the KOG annotations and the analysis given above, we identified four major pathways: phenylalanine metabolism (ko00360), phenylpropanoid biosynthesis (ko00940), starch and sucrose metabolism (ko00500), and pentose and glucuronate interconversion (ko00040). The metabolic pathways associated with these pathways could facilitate further analyses of the genes and molecular mechanisms associated with fertility restoration in K-TCMS lines.

### Identification of transcription factors related to fertility restoration

**Fig. 7:**
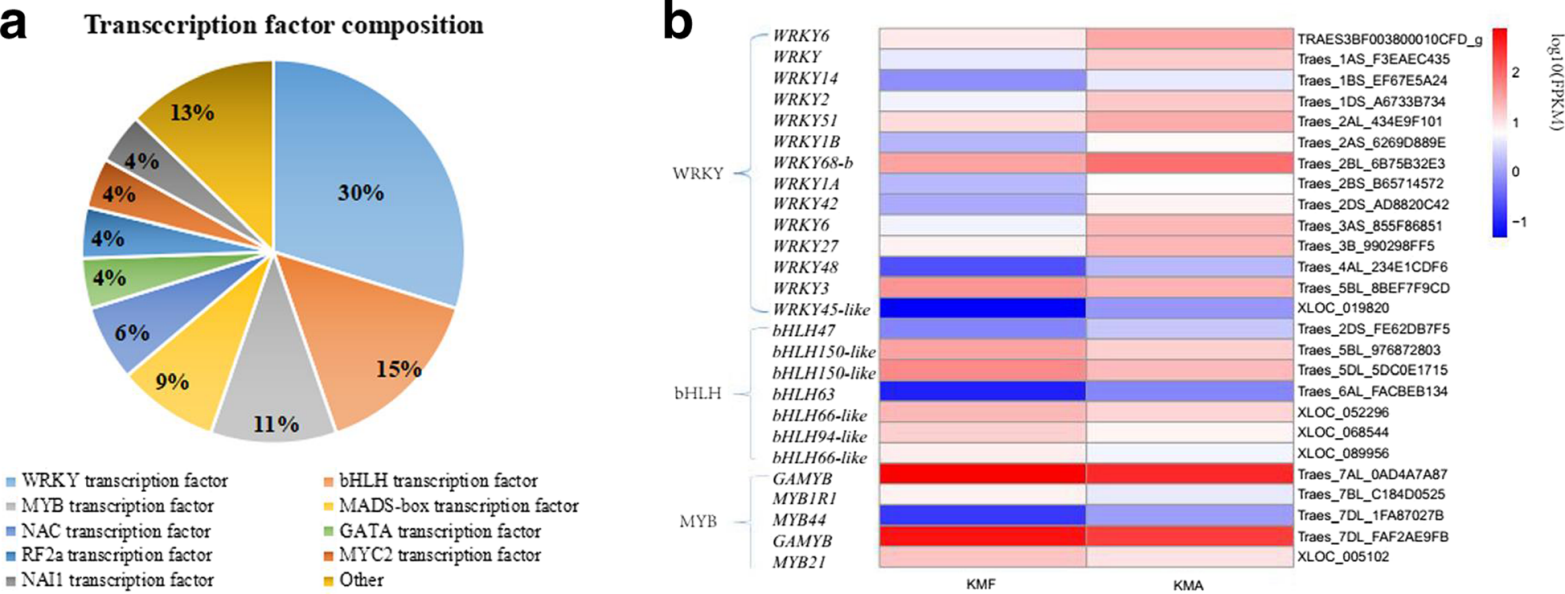
Analysis of transcription factors. Relative proportions of different transcription factors detected (**a**). Heat map showing the expression levels of WRKY, bHLH, and MYB transcription factors in KMF and KMA (**b**). The log10 (FPKM) value is shown by the color gradient from low = blue to high = red

The regulation of plant gene expression comprises a complex network. Transcription factors can regulate structural genes related to specific traits, and thus their effects on plant growth and development may be greater than those of structural genes. Based on previous studies of transcription factors [25–27] and the annotation information obtained from the Nr database (ftp://ftp.ncbi.nlm.nih.gov/blast//db/FASTA/), 47 DEGs were predicted to encode transcription factors, which were divided into nine families comprising WRKY, bHLH, MYB, MADS-box, NAC, GATA, RF2a, MYC2, and NAI1 transcription factors. The WRKY transcription factors were most common and they were encoded by 14 DEGs, which accounted for 30% of the total, followed by bHLH transcription factors (seven DEGs, 15%) and MYB transcription factors (five DEGs, 11%) (Fig. 7a). Analyses of the expression levels of these three major transcription factor families in KMF and KMA detected similar trends (Fig. 7b, Additional file 6: Table S6). The heat map showed that excluding the upregulation of *WRKY3* in KMF compared with KMA, the other 13 DEGs were downregulated. *bHLH47*, *bHLH63*, and *MYB44* were downregulated in KMF, but the remaining transcription factors were upregulated, i.e., bHLH and MYB. These findings suggest that WRKY, bHLH, and MYB family transcription factors may affect fertility restoration in K-TCMS lines.

### Carbohydrate metabolism and Phenylpropanoid biosynthesis pathways involved with the regulation of pollen development with *Aegilops kotschyi* cytoplasm

**Fig. 8:**
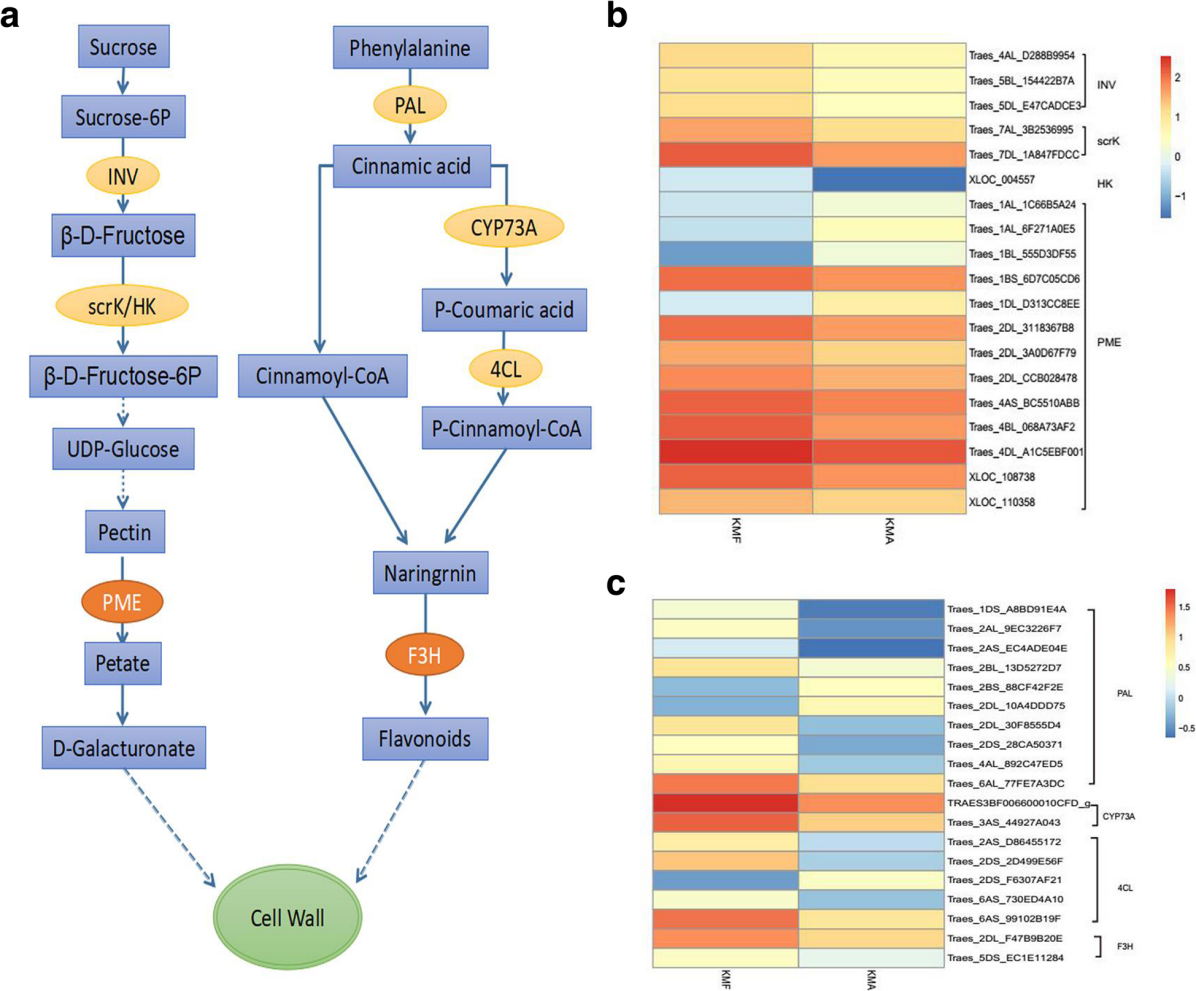
Carbohydrate metabolism and phenylpropanoid biosynthesis pathways (**a**). Heat map showing the expression levels of some differentially expressed genes (DEGs) encoding enzymes in the carbohydrate metabolic pathway in KMF and KMA (**b**). Heat map showing the expression levels of some DEGs encoding enzymes in the phenylalanine metabolism and biosynthetic pathways in KMF and KMA (**c**). The log10 (FPKM) value is shown by the color gradient from low = blue to high = orange

According to the analyses based on KOG and KEGG, a large number of DEGs were mainly enriched in the carbohydrate metabolism and phenylpropanoid biosynthesis and metabolic pathways. To clarify whether these DEGs regulate pollen development via these pathways, we mapped the metabolic pathways regulated by some of the key DEGs in the four key pathways identified previously (Fig. 8a). In the pollen maturation process, sugars provide the basis for pollen growth and development. In the sugar metabolism process, sucrose is used as a substrate to produce sucrose 6-phosphate and β-D-fructose is produced under catalysis by hydrolase beta-fructofuranosidase (INV, EC: 3.2.1.26). β-D-Fructose is then used to produce β-D-Fructose-6P under catalysis by transferase fructokinase (scrK, EC: 2.7.1.4) and hexokinase (HK, EC: 2.7.1.1), which is catalyzed by a series of enzymes to finally produce uridine diphosphate-glucose (UDP-glucose). UDP-glucose is an important intermediate in monosaccharide interconversion or the formation of uranic acid, and it plays a central role in carbohydrate metabolism. UDP-glucose is catalyzed by a series of enzymes to produce pectin. Pectin is hydrolyzed by pectin methylesterase (PME, EC: 3.1.1.11) to form D-galacturonate and maintain cell wall development. The expression levels of these key enzymes in fertile and sterile anthers were shown in the heat map in Fig. 8b (Additional file 7: Table S7). Traes_1AL_1C66B5A24, Traes_1AL_6F271A0E5, Traes_1BL_555D3DF55, and Traes_1DL_D313 CC8EE were downregulated in fertile anthers but the other enzymes encoded by DEGs were upregulated in fertile anthers and downregulated in infertile anthers. Therefore, we hypothesized that the upregulated expression of these enzymes can maintain normal pollen growth and development. It should be noted that many DEGs encoded PMEs with roles during sugar metabolism. In the phenylpropanoid metabolism and biosynthetic pathway, phenylalanine is used as a substrate to form cinnamic acid under the action of phenylalanine ammonia-lyase (PAL, EC: 4.3.1.24). Cinnamic acid finally produces P-cinnamoyl-CoA under the actions of oxidase trans-cinnamate 4-monooxygenase (CYP73A, EC: 1.14.13.11) and 4-coumarate-CoA ligase (4CL EC: 6.2.1.12). P-Cinnamoyl-CoA and cinnamoyl-CoA produce naringenin, which finally yields flavonoids under catalysis by oxidoreductase flavanone 3-hydroxylase (F3H, EC: 1.14.11.9), which maintains the development of the pollen tubes and pollen walls. The heat map showing the expression levels of these key enzymes is presented in Fig. 8c (Additional file 7: Table S7). Traes_2BS_88CF42F2E, Traes_2DL_10A4DDD75, and Traes_2DS_F6307AF21 were downregulated in fertile anthers, but the other key enzymes encoded by these DEGs were upregulated in fertile anthers. These results indicate that the upregulation of carbohydrate metabolism and phenylpropanoid biosynthesis as well as metabolism-related genes in fertile (KMF) anthers may ensure the normal development and fertility of pollen.

### Total soluble sugar and flavonoid contents

**Fig. 9:**
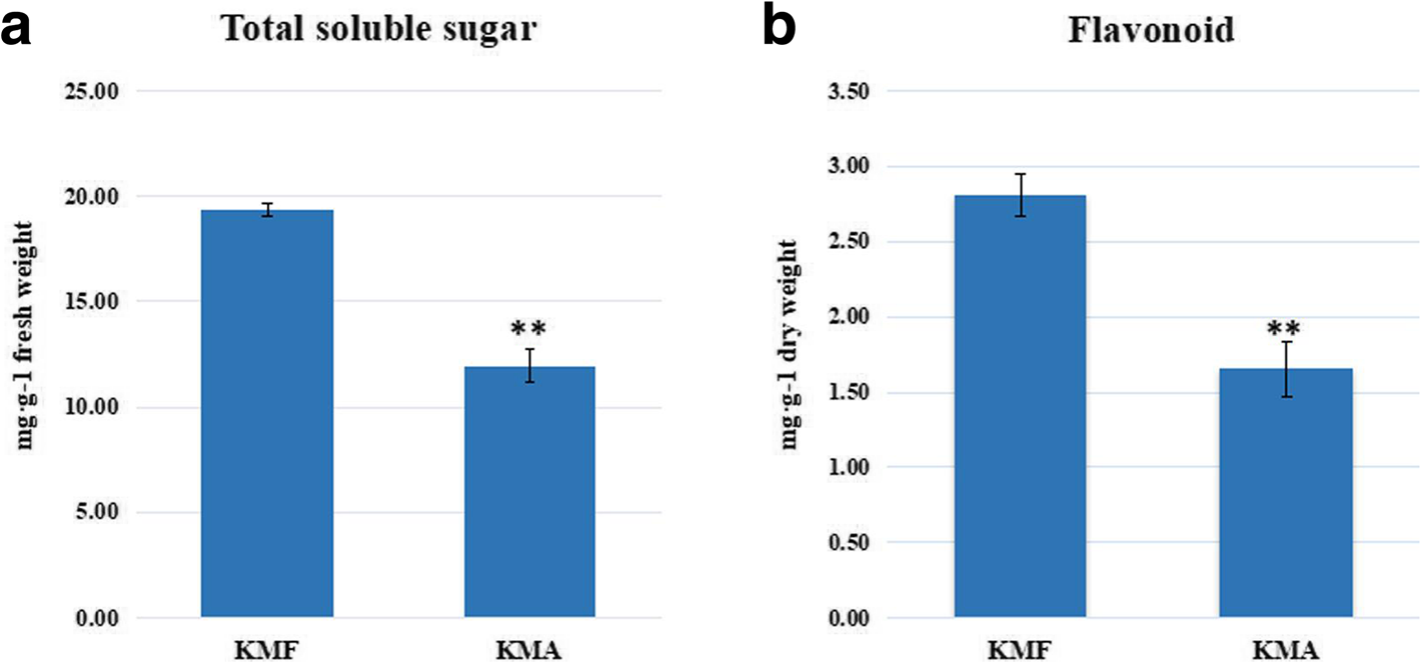
Total soluble sugar and flavonoid contents in the anthers from KMF and KMA. Total soluble sugar contents (**a**). Flavonoid contents (**b**). Data represent the mean and standard deviation based on three replicates. ** *p* < 0.01 according to the Student’s t-test

The soluble sugar and flavonoid contents were determined to elucidate the changes in carbohydrate metabolism and phenylpropanoid biosynthesis and metabolism in the fertile anthers (KMF) and sterile anthers (KMA). In the carbohydrate metabolism pathway, most of the DEGs were upregulated in KMF. Thus, we hypothesized that the expression of genes related to fertility may lead to differences in the total soluble sugar contents in fertile (KMF) and sterile (KMA) plant anthers, where the fertile plant anthers may have higher sugar contents. Therefore, we measured the total soluble sugar contents in the anthers from KMF and KMA plants, and the results showed that the soluble sugar contents of the fertile (KMF) anthers were significantly higher than those of the sterile (KMA) anthers (Fig. 9a, Additional file 8: Table S8). Moreover, previous studies have shown that phenylpropanoid metabolism has important links with flavonoids and it influences anther development [28]. We found that F3H was highly expressed in fertile (KMF) anthers but barely expressed in the sterile (KMA) anthers according to the RNA sequencing results, and thus we measured the flavonoid contents in anthers from KMF and KMA plant. The results showed that the flavonoid contents of the fertile (KMF) anthers were significantly higher than those of the sterile (KMA) anthers (Fig. 9b, Additional file 9: Table S9). These results agreed with our hypothesis and also verified the reliability of the sequencing results.

### Verification of DEGs by qRT-PCR

**Fig. 10:**
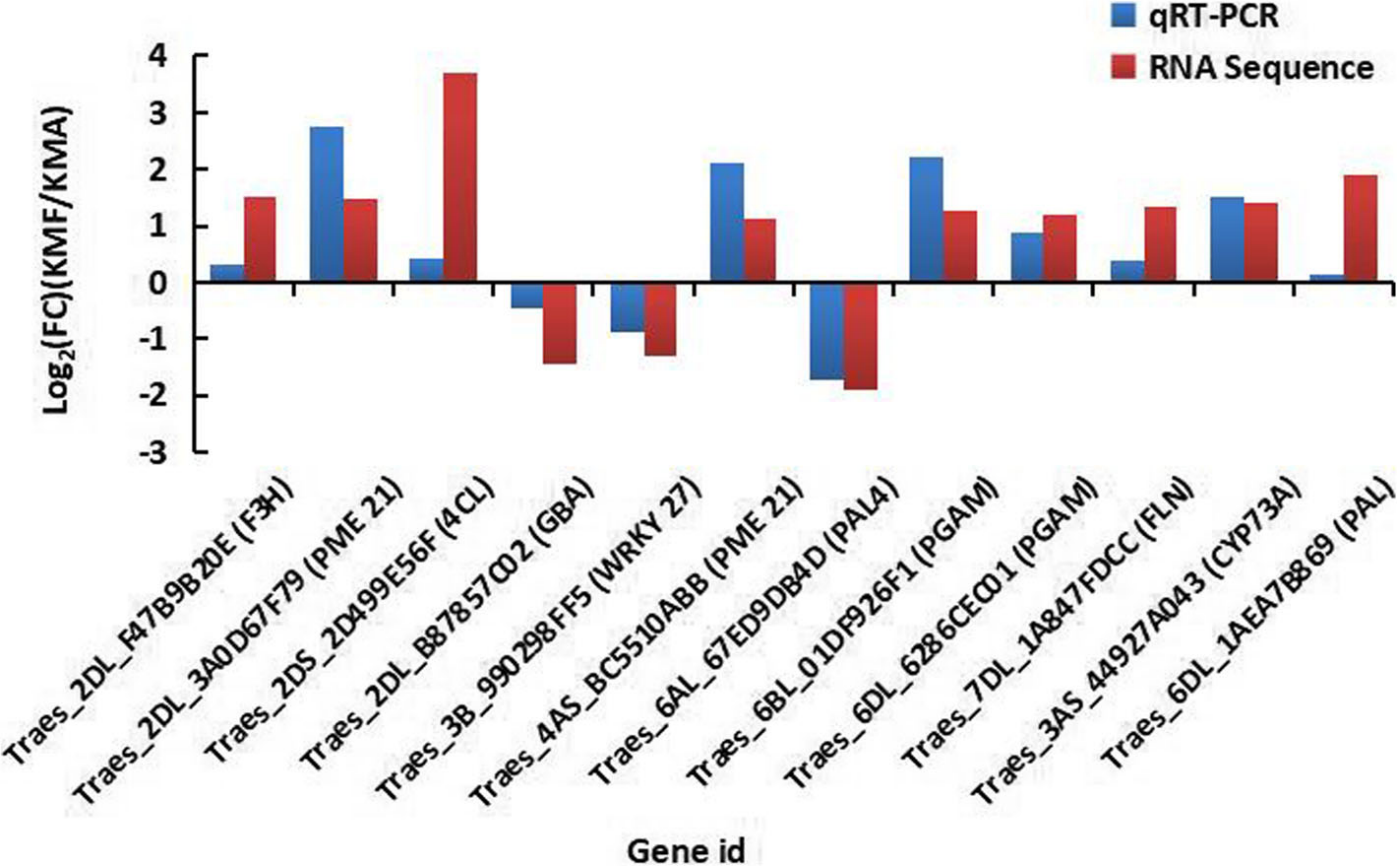
qRT-PCR validates the RNA sequencing results of some DEGs. Data obtained by qRT-PCR represent means based on three replicates. Log2 (FC) represents logarithmic value of change in expression for KMF relative to KMA

In order to validate the differential expression patterns detected for DEGs by RNA sequencing, 12 DEGs were selected for qRT-PCR detection from the significant metabolic pathways, which encoded F3H (Traes_2DL_F47B9B20E), pectinesterase/pectinesterase inhibitor 21 (PME 21, Traes_2DL_ 3A0D67F79, Traes_4AS_BC5510ABB), 4-coumarate: coenzyme A ligase (4CL, Traes_2DS_ 2D499E56F), lysosomal beta glucosidase (GBA, Traes_2DL_B87857C02), WRKY27 transcription factor (WRKY 27, Traes_3B_990298FF5), phenylalanine ammonia-lyase 4 (PAL 4, Traes_6AL_ 67ED9DB4D), 2,3-bisphosphoglycerate-dependent phosphoglycerate mutase (PGAM, Traes_6BL_ 01DF926F1, Traes_6DL_6286CEC01), fructokinase-2-like (FLN, Traes_7DL_1A847FDCC), trans-cinnamate 4-monooxygenase (CYP73A, Traes_3AS_44927A043), phenylalanine ammonia-lyase (PAL, Traes_6DL_1AEA7B869). The expression patterns of these genes are shown in Fig. 10 (Additional file 10: Table S10), which demonstrated that the gene expression patterns obtained by qRT-PCR and RNA sequencing data exhibited similar trends, thereby confirming the accuracy of the RNA sequencing results obtained in this study.

## Discussion

### Carbohydrate metabolism affects Cell Wall development, anther growth, and fertility

From the differentiation of the pistil and stamen until the formation of the mature pollen grains, plants synthesize and degrade materials frequently in order to provide energy to support pollen germination, pollen tube growth, and fertilization. Carbohydrates provide energy to maintain the development of the anthers and pollen, but they also act as signals that influence their development [29]. A large number of related genes or proteins are involved in the combined regulatory process. Many studies have shown that the abnormal expression of genes or proteins in anthers interferes with pollen development, thereby affecting pollen fertility. PME (E3.1.1.11) belongs to a large family of carbohydrate esterases and this enzyme regulates the degree of pectin methylation as well as being a major component of plant cell walls. Previous studies of the function of PME indicate that it is involved in cell wall loosening and it also participates in various plant growth processes, such as pollen formation and pollen tube elongation [30]. PME can endogenously regulate the pectin content between plant cell walls and cells. After demethylation by PME, pectic acid binds to Ca^2+^ to form insoluble substances, which inhibit cell separation and organ shedding [31]. The loss of function by VANGUARD1 may reduce the efficiency of the interaction between the pollen tube and the extracellular matrix in the style and transmitting tract, thereby leading to the blockage of the pollen tube in the style and transmitting tract to greatly reduce male fertility [32]. *BcMF23a* and *BcMF23b* are expressed in microspores during the dinuclear pollen and mature pollen stages, and they are highly expressed in fertile flower buds, but silent in genic male sterile lines [33]. In the starch and sucrose metabolism pathway, PME catalyzes the hydrolysis of pectin to produce pectic acid and methanol. In the present study, 13 DEGs were annotated as PMEs and nine were significantly upregulated in fertile anthers but not in infertile anthers (Fig. 8b, Additional file 7: Table S7), and the other four had very low expression levels in both fertile and sterile anthers. These DEGs were annotated in the starch and sucrose metabolism, and pentose and glucuronate interconversion pathways. It is notable that of the 14 DEGs detected in the pentose and glucuronate interconversion pathway, 13 encoded PMEs. Therefore, we suggest that PME enzymes have crucial effects on cell wall development, anther growth, and fertility, and thus the roles of PMEs in wheat fertility merit further study.

### Flavonoids are crucial for the development of fertile anthers

Many studies have shown that the phenylalanine metabolism and biosynthetic pathways have important roles in plant fertility. During the pollen maturation process, phenylalanine is used as a substrate for a series of biochemical enzymes, including PAL, C4H (Cinnamic acid 4 hydroxylase), CHS (Chalcone synthase;), CHI (Chalcone isomerase), and FLS (Flavonol synthase), before the formation of phenolic resin and long-chain fatty acids, and sporopollenin is finally synthesized as the main component of the pollen wall [34]. Defects in the pollen wall structure can lead to abnormal pollen development, which affects the fertility of plants [35]. In the present study, numerous DEGs were enriched in the phenylpropanoid metabolism and biosynthetic pathways. The enzymes encoded by these DEGs were upregulated in fertile anthers and we suggest that they may have important relationships with fertility. Flavonoids are synthesized via the phenylpropane pathway [36] and they have key roles in pollen development because they are essential for pollen maturation and pollen tube growth in flowering plants [37]. In addition, F3H is a key enzyme for the formation of flavonoids and it belongs to the family of dioxygenases that depend on 2-ketoglutarate [38]. F3H can regulate metabolism alone but it often co-catalyzes the synthesis of flavonoids with chalcone isomerase and chalcone synthase [39]. In the present study, F3H was upregulated in fertile pollen but its expression level was low in infertile anthers. Flavonoids are the main raw material used for the synthesis of pink pigments as well as being important for the development of normal anthers. We also detected high levels of flavonoids in fertile anthers, thereby supporting the key role of flavonoids in fertility.

### Transcription factors involved are in the regulation of pollen development and male fertility

In flowering plants, male gametophytes (or pollen grains) play a vital role in plant fertility, and the development of pollen grains is strictly controlled, where it involves the complex and precise regulation of many transcription factors [40, 41]. Changes in the expression levels of some transcription factors may lead to abnormal pollen development and male sterility during plant growth and development. For example, the overexpression of *WRKY27* in *Arabidopsis* plants leads to pollen cracking defects, growth aberrations, and male sterility [42]. *WRKY2* and its close homolog *WRKY34* are required for male gametogenesis and they play important roles in pollen development and function. Thus, the overexpression of *WRKY 34* in mature pollen from transgenic *Arabidopsis* plants was associated with greatly reduced fertility [40, 43]. Previous studies have also shown that many WRKY transcription factors are linked with resistance to abiotic and biotic stresses. However, the effects of WRKY on the control of pollen and flower growth and development have not been widely investigated, and previous studies have focused mainly on *Arabidopsis*. In the present study, we detected 14 genes encoding WRKY transcription factors that were specifically expressed in wheat anthers with differences in fertility, i.e., *WRKY6*, *WRKY*, *WRKY14*, *WRKY2*, *WRKY51*, *WRKY1B*, *WRKY68-b*, *WRKY1A*, *WRKY42*, *WRKY27*, *WRKY48*, *WRKY3*, and *WRKY45-like*. The expression levels of 13 of these WRKY transcription factors were upregulated in male sterile wheat anthers (Fig. 8b), thereby indicating that their upregulation in sterile wheat has important effects on fertility. Further studies of the regulation of these WRKY transcription factors in wheat pollen grains may enhance our understanding of the molecular mechanisms related to pollen development and fertility.

The MYB transcription factor family is a large class of transcription factors and they are also involved in the regulation of gene expression during plant growth, where they mainly participate in primary and secondary metabolism, including phenylpropanoid metabolism [44] and flavanol biosynthesis [45]. Moreover, some MYB transcription factors are associated with flower development. The R2R3-MYB transcription factor MYB21 plays a dominant role in the elongation of stamen filaments. In *Arabidopsis*, the overexpression of *MYB21* partially restores stamen filament growth and fertility in the *opr3* mutant [46, 47]. Gibberellin MYB is involved with gibberellic acid signal transduction, which strongly affects flower development in angiosperms, but mainly anther and pollen growth [48]. In addition, some bHLH transcription factors interact with MYB transcription factors to regulate diverse plant responses, including secondary metabolic processes, flavonoid pathways, and stamen development [49]. For example, bHLH transcription factors (MYC2, MYC3, MYC4, and MYC5) interact with MYB transcription factors (MYB21 and MYB24) to form the bHLH-MYB complex, and jasmonates repress the bHLH-MYB complex to regulate jasmonic acid-mediated stamen development and fertility [50]. Some modulators of flavonoid biosynthesis that interact with R2R3-MYBs or bHLHs can promote or impair the production of flavonoids [51]. In our study, most of the bHLH and MYB transcription factors were upregulated in fertile wheat and downregulated in sterile wheat (Fig. 8b). The variable expression levels of these MYB and bHLH transcription factors suggest that they have relationships with wheat fertility and they may have important roles in this process. These results provide novel insights into flavonoid biosynthesis and fertility, thereby increasing our understanding of the various mechanisms responsible for regulating fertility.

### Mitochondria-associated genes affecting pollen fertility restoration

The normal development of plant pollen is inseparable from the coordinated interaction between nucleus and cytoplasm, and is inseparable from the selective expression of genes.

Studying mitochondria-related gene expression is of great significance for understanding the temporal and spatial expression of genes and elucidating the molecular mechanism of cytoplasmic male sterility. Mitochondria is a semi-autonomous organelle that encodes its own rRNA, tRNA and a small amount of protein, and it is inseparable from the guidance and regulation of the nuclear genome [52]. Studies have shown that pollen fertility restoration is a process of nucleo-cytoplasmic interaction. When one or more fertility restoration genes are introduced, the structure or expression of CMS-related genes are regulated accordingly, so that the function of mitochondria returns to normal, thus making pollen fertility is restored [53]. Some studies have found that the normal expression of mitochondria-related genes plays an important role in pollen development and fertility. The Mg^2+^ transporter *MRS2/MGT* gene family mainly mediates magnesium transport between cytoplasm and mitochondria [54]. Studies have found that *AtMGT 5* gene is specifically expressed in anthers at early flower development, and male gametes of *AtMGT 5* mutant produce defective pollen [55]. In addition, disruption of *AtMGT 9* gene expression leads to abortion of mature pollen grains [56], indicating *AtMGT 9* and *AtMGT 5* genes are critical in pollen development and male fertility. In this study (Additional file 11: Table S11), two genes annotated as the mitochondrial inner membrane magnesium transporter mrs2 were up-regulated in fertile anthers, which expressed twice as much as the sterile anthers, indicating normal expression of the mrs2 gene is associated with fertility restoration of fertile anthers. NADH dehydrogenase is an enzyme that catalyzes the transfer of electrons from NADH to Coenzyme Q in the mitochondrial inner membrane, and its presence makes the plant’s respiratory chain more flexible in the process of bioenergy function [57]. Formate dehydrogenase (FDH) is a NAD+ dependent enzyme widely distributed in organisms, and FDH catalyzes the oxidation of formic acid to carbon dioxide while reducing NAD+ to NADH in this process [58]. ATP is the main energy currency of cells, which is catalytically decomposed into ADP and inorganic phosphate by ATPase to promote most biological reactions in the cytoplasm. Mohammed Sabar et al. found that the ATPase activity of F1-F0-ATP synthase in the sterile line was significantly lower than that of the fertile line by comparing the gelase activities of several mitochondrial respiratory complexes between the fertile line and the sterile line, and thought that this may be the effect of *orf 522* expression, which makes the sterile line unable to meet the energy requirements needed to maintain anther development, leading to pollen abortion [59]. In our study, two genes encoding NADH dehydrogenase, one encoding formate dehydrogenase and five encoding AAA-ATPase were down-regulated in sterile wheat anthers. We predicted that the down-regulated expression of this series of related enzymes affects energy transfer in mitochondria associated with cytoplasmic and nuclear interactions, which in turn affects fertility restoration of pollen. These findings provide new insights into the potential molecular regulation mechanisms of mitochondria-associated genes and fertility restoration in cytoplasmic male sterile wheat.

## Conclusions

The induction of fertility restoring genes in the presence of *Aegilops kotschyi* cytoplasm causes normal pollen development and fertility in KTM3315R, mainly via effects on carbohydrate metabolism, phenylpropanoid metabolism and biosynthesis, and upregulating the expression of candidate genes that encode PME and F3H, which are closely related to pollen development. In addition, the regulation of pollen development and male fertility is affected by the WRKY, bHLH, and MYB transcription factor families, where male fertility is associated with the downregulation of WRKY and the upregulation of bHLH and MYB. Thus, these transcription factors can enhance pollen growth and development, and restore male fertility in wheat with *Aegilops kotschyi* cytoplasm. Our results indicate that the genes responsible for regulating fertility restoration play important roles in the presence of *Aegilops kotschyi* cytoplasm, thereby providing a basis for exploring the molecular mechanisms that regulate changes in wheat fertility.

## Methods

### Plant materials

The study materials comprised the near-isogenic lines KTM3315A (designated as KMA, a K-TCMS line with *Aegilops kotschyi* cytoplasm) and KTM3315R (designated as KMF, a cytoplasmic male fertile line with *Aegilops kotschyi* cytoplasm), which were developed over the course of many years by Northwest A&F University. The sterile line KTM3315A was used as a female parent to hybridize with the homologous maintainer line TM3315B for which anthers are provided to produce the sterile line KTM3315A (sterile line propagation method). The breeding procedure of KTM3315R is as follows: Chinese Spring has a high and stable restoration degree (seed-setting rate, ~ 75.00%) for K-CMS [34], so we selected restorer Chinese Spring as the pollen donor hybridized with KTM3315A to produce F_1_ generation of KTM3315A and Chinese Spring. K-CMS lines belong gametophytic male-sterile type [60] and the recessive genes could hardly be transmitted by male gametes (~ 0.54%), so we selected its homologous maintainer TM3315B as the pollen donor and crossed it with the F_1_ generation of KTM3315A and Chinese Spring. As a recurrent parent, TM3315B was backcrossed 8 times with the F_1_ generation, and the fertile plants were selected to produce the BC_8_F_1_ population. The BC_8_F_1_ population was self-crossed for 5 generations, forming a near-isogenic line KTM3315R that was stably inherited. The breeding procedure is illustrated in Additional file 12: Figure S1.

During October 2016, KMF and KMA were cultivated under natural conditions and regular field management at the Northwest A&F University experimental station in Yangling (34°15′N, 108°08′E), China. During May 2017, three spikes were selected randomly on each plant for self-pollination and bagged at the heading stage until subsequent fertility analyses. The stages and fertility of the other spikes were identified using 1% acetocarmine and 1% I_2_–KI. Anthers from the binucleate stage were collected as three biological replicates for 20 fertile plants and 20 sterile plants with equal amounts from KMF and KMA for sequencing. The fertile and sterile anthers were immediately snap frozen in liquid nitrogen and stored at − 80 °C until use.

### Phenotypic traits and microscopic observations

Photographs of the sterile and fertile wheat anther phenotypes were obtained using a Nikon E995 digital camera (Nikon, Tokyo, Japan) mounted on a Motic K400 dissection microscope (Preiser Scientific, Louisville, KY, USA). The different anther development stages were identified by staining with 1% acetocarmine [61]. To evaluate the viability of mature pollen grains, anthers in the stage before dehiscence were crushed in 1% I_2_–KI, incubated for 15 to 20 min at room temperature with I_2_–KI, washed with buffer, and the pollen grains were then observed by microscopy [62]. The anthers, anther outer epidermis, and trinucleate stage microspores were analyzed by scanning electron microscopy as described by [63] with a JSM-6360LV scanning electron microscope (JEOL, Tokyo, Japan).

### RNA extraction, cDNA library construction, and Illumina deep sequencing

The total RNA was extracted from six samples of KMF and KMA plant anthers, each with three biological replicates, according to the instruction manual provided with the RNAiso for Polysaccharide-rich Plant Tissue kit (Takara Biological Engineering (Dalian) Co. Ltd., China). The RNA concentration and purity were measured using a NanoDrop 2000 Spectrophotometer (Thermo Fisher Scientific, Wilmington, DE, USA). RNA integrity was assessed using an RNA Nano 6000 Assay Kit for the Agilent Bioanalyzer 2100 System (Agilent Technologies, Inc., Santa Clara, CA, USA). After the RNA samples passed through these three steps, eukaryotic mRNA was enriched with Oligo (dT) beads. The enriched mRNA was then fragmented into short fragments using fragmentation buffer and reverse transcribed into first-strand cDNA with random primers. Second-strand cDNA was synthesized using DNA polymerase I, RNase H, dNTP, and buffer. The cDNA samples were purified with a QIAquick Gel Extraction kit (Beijing Lanbo Kangsi Technology Co. Ltd., China), before end repair, adding poly (A) tails, and ligating to Illumina sequencing adapters. The ligation products were selected by size via agarose gel electrophoresis, before PCR amplification, and sequencing using the Illumina HiSeq™2500 system by Sagene Biotech Co. Ltd. (Guangzhou, China).

### RNA sequencing data analysis

The reads obtained by sequencing comprised raw reads containing adapters or low quality bases, which would have affected the subsequent assembly and analysis steps. Thus, the raw reads were filtered to obtain high-quality reads by removing low-quality reads containing adapters, unknown nucleotides (*N* > 10%), and more than 50% low quality bases (Q-value ≤20). The short reads alignment tool Bowtie2 [64] was used to map the reads to the ribosomal RNA (rRNA) database (ftp://ftp.ncbi.nlm.nih.gov/genbank/). The reads that mapped to the rRNA database were removed. The remaining reads from each sample were mapped to the Ensembl release 31 IWGSC1.0 + NC_002762.1 reference genome with TopHat2 (version 2.0.3.12) [65]. The transcripts were reconstructed with Cufflinks (version 2.2.1) [66]. Gene abundances were quantified with RSEM (version 1.2.31) [67]. The gene expression levels were normalized using the fragments per kilobase of transcripts per million mapped reads (FPKM) method.

### Bioinformatics analysis of DEGs

DEGs were functionally annotated using the non-redundant protein database (Nr; NCBI: ftp://ftp.ncbi.nlm.nih.gov/blast//db/FASTA/). The DEGs were then further aligned with the Clusters of Orthologous Groupss (https://www.ncbi.nlm.nih.gov/COG/) database to predict and classify their functions [68], before enrichment analysis according to their Gene Ontology (GO; http://www.geneontology.org/) functions and Kyoto Encyclopedia of Genes and Genomes) (KEGG; http://www.genome.jp/kegg/genes.html) pathways. In addition, the GO annotations were analyzed to identify the main biological functions of the DEGs where the Blast2GO program [69] (https://www.blast2go.com/) was used to obtain GO annotations for all of the DEGs. The results were submitted to WEGO (http://wego.genomics.org.cn) to generate a GO classification graph for all of the DEGs. KEGG is the major public database related to pathways [70] and it can identify the major biochemical metabolic pathways and signal transduction pathways for DEGs. According to the GO and pathway enrichment analysis results, q-value ≤0.05 was selected as the threshold of significance to determine enrichment in the DEG sets [71]. OmicShare small tools2 (http://www.omicshare.com/tools/) was employed to obtain a heat map without rows and column clusters.

### Assays of soluble sugar and flavonoid contents

Approximately 0.1 g of the fertile (KMF 1, KMF 2, and KMF 3 represents three biological repeats, respectively, the same as following) or sterile (KMA 1, KMA2, and KMA3) anthers was weighed, before adding 1 mL of distilled water, and grinding into a homogenate, which was then poured into a 2 mL centrifuge tube and the lid was closed. The sample was heated in a water bath at 95 °C for 10 min, cooled, and then subjected to centrifugation at 8000×g and 25 °C for 10 min, before pouring the supernatant into a 10 mL test tube. The volume was adjusted to 10 mL with distilled water, before shaking gently by hand for about 30 s. The procedures and calculations were performed according to the instructions provided with a Plant Soluble Sugar Content Determination kit (Suzhou Keming Biotechnology Co. Ltd., China).

The flavonoid contents were determined by drying samples (same as above, KMF 1, 2, 3 and KMA 1, 2, 3) to a constant weight and crushing after passing through a 40-mesh sieve. Approximately 0.1 g of the crushed sample was weighed and added to 2.5 mL of distilled water, before ultrasonic extraction (ultrasonic power = 300 W, crushing = 5 s, intermittent = 8 s, temperature = 60 °C, extraction = 30 min). The sample was then subjected to centrifugation at 12000 rpm and 25 °C for 10 min, before analyzing the supernatant from the extract. A spectrophotometer was turn on for more than 30 min and the wavelength was adjusted to 502 nm, before zeroing with distilled water and measuring the absorbance of each sample. The operations and calculations were performed according to the instructions provided with a Plant Flavonoid Content Determination kit (Sino Best Biological Technology Co., Ltd., China).

### Confirmation of candidate DEGs by quantitative reverse-transcription PCR (qRT-PCR)

To validate the DEGs detected by RNA sequencing, 12 DEGs were selected from the significant metabolic pathways. The primers used for qRT-PCR were designed with Primer-BLAST (https://www.ncbi.nlm.nih.gov/tools/primer-blast/) and synthesized by Xi’an Qingke Zexi Biotechnology Co. Ltd., China. The actin gene (GenBank: GQ339766.1) was used as a reference to normalize the gene expression levels where it was set to 1 [72]. qRT-PCR analysis was performed as described by Ye [35]. The sequence-specific primer pairs used for qRT-PCR are listed in Additional file 13: Table S12, with those for the actin gene and the 12 selected DEGs. The analysis was conducted using three technical replicates for each sample. We calculated the expression level of the actin gene using the 2 ^− ΔΔCt^ method and normalized the relative expression levels with respect to the expression level of the actin gene [73].

## Additional files

Additional file 1: **Table S1.** Summary of sequencing results. (XLSX 10 kb)

Additional file 2: **Table S2.** Differentially expressed genes. (XLSX 387 kb)

Additional file 3: **Table S3.** Gene Ontology annotations. (XLSX 66 kb)

Additional file 4: **Table S4.** KOG class annotations. (XLSX 30 kb)

Additional file 5: **Table S5.** Significantly enriched KEGG pathways. (XLSX 14 kb)

Additional file 6: **Table S6.** WRKY, bHLH, and MYB transcription factors. (XLSX 12 kb)

Additional file 7: **Table S7.** DEGs involved in pathways. (XLSX 17 kb)

Additional file 8: **Table S8.** Soluble sugar contents. (XLSX 11 kb)

Additional file 9: **Table S9.** Flavonoid contents. (XLSX 11 kb)

Additional file 10: **Table S10.** The gene expression of qRT-PCR and RNA sequencing. (XLSX 15 kb)

Additional file 11: **Table S11.** Mitochondrial function-related genes. (XLSX 14 kb)

Additional file 12: **Figure S1.** Breeding procedure of near-isogenic lines. (DOCX 1939 kb)

Additional file 13: **Table S12.** Sequence-specific primers used for qRT-PCR. (XLSX 11 kb)

## Abbreviations

4CL: 4-Coumarate-CoA ligase
CMS: Cytoplasmic male sterility
CYP73A: Trans-cinnamate 4-monooxygenase
DEGs: Differentially expressed genes
F3H: Flavanone 3-hydroxylase
FC: Fold Change
FDR: False Discovery Rate
FPKM: Fragments Per Kilobase of transcript per Million mapped reads
GBA: lysosomal beta glucosidase
HK: Hexokinase
INV: Hydrolase beta-fructofuranosidase
KEGG: Kyoto Encyclopedia of Genes and Genomes
KMA: KTM3315A
KMF: KTM3315R
KOG: Eukaryotic Orthologous Groups
K-TCMS: Thermo-sensitive male-sterility
PAL: Phenylalanine ammonia-lyase
PGAM: 2,3-bisphosphoglycerate-dependent phosphoglycerate mutase
PME: Pectin methylesterase/pectinesterase
scrK: Fructokinase
UDP-glucose: Uridine diphosphate-glucose
