# Supplementary figures and images for: Analysis of metabolic pathways related to fertility restoration and identification of fertility candidate genes associated with Aegilops kotschyi cytoplasm in wheat (Triticum aestivum L.)
Source: BMC Plant Biol. 2019 Jun 11;19:252. doi: 10.1186/s12870-019-1824-9 (PMC6560861; doi:10.1186/s12870-019-1824-9)

**Figure S1** Breeding procedure of near-isogenic lines


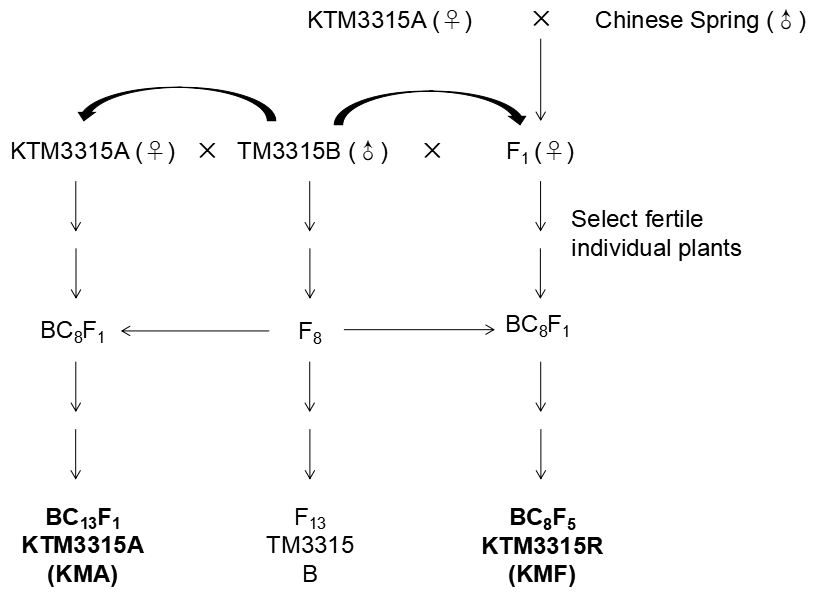

Supplement: Supplementary file 12 — Figure S1. Breeding procedure of near-isogenic lines. (DOCX 1939 kb) [file 12870_2019_1824_MOESM12_ESM.docx]
